# Event and Comment

**Published:** 1920-12

**Authors:** 


### DENTAL REGISTER

THE MAGAZINE OF DENTISTRY

Vol. LXXIV DECEMBER, 1920 No. 12

### EVENT AND COMMENT

**What's the Matter with Dentistry?** Many dental educators, writers and practitioners seem to think something more is required of dentists, colleges, and students than was expected in other times. We are asking for more dentists, better educators, more skillful practitioners. Modern dentistry is new and yet we are demanding the learning and science of an accumulated century. Our art has developed so quickly that its growth is considered so vigorous that at times it might be characterized as rank. We are not therefore suprised or peeved when some stately critic calls us to account for a supposed impertinence. While we are conscious of imperfections and because of our numerous errors in judgment, we do these things unconsciously and we mean no impertinence. In about so often some one writes to our professor, or our editor, or sends it to our journal, and tells us a lot of disagreeable things that may not be real mean, but just the same we don't like what is said and we wish the people knew enough to keep still when they are faultfinding or criticising. Sometimes these criticisms are aimed at our college, or ourselves, and they are generally uncalled for. We enjoy real honest differences when they make one think and they really make us grow and do us a lot of good. When they come at us with intent to hurt or terrify us we feel like resenting the attack the best we know how.

Recently an eminent author, a teacher, a scientist, a specialist in dental practice, and with it all a good fellow with most kindly intentions, said a lot of things in a popular dental magazine that didn't look nice to us when they were printed. We realize that much of what he said could be contravened. We can not see how it would be practicable at the present time to change the present practices to accord with the ideas he advocates. To illustrate, he characterizes a method of practice as obsolete, and said its teaching had "retrogressed rather than advanced." This may have been a fact in this particular instance, and may have been a failure from the standpoint of a former time, or as a method utilized as a variant form of manipulation. Methods have been approved for years only to be discredited experience and again be affirmed by later experiences by others. There are only a few methods and practices that have in a large measure been certified as standard, because we are new, and are in the period of certification, as it were, at this time. Our knowledge, our education, and our ideals in fact are as yet in a state of fluxion. Our technical procedures, even our nomenclature, are undergoing changes and readjustments that make it difficult to standardize dentistry and evaluate it in terms of either dental or medical science. The two sciences have much that is common but their arts are as different as may be from each other.

Our critic can not seem to recognize that dentistry as a science is vitally an integral part of medicine, and it ever has been, but that the art of dentistry never has been more than an associate in the practice of medicine. Dentistry has no controversy with medical practice, and while there is much that dentists would like to know about the art of medical practice because of our intimate relations it does not seem that the chasm in these arts is growing less wide as time goes on. It seems improbable that dentistry will ever become a specialty of medicine in affiliation or as a specialty even of the medical art. If this is so why should we spend so much time in building an educational system that meets only the needs of the medical practitioner's ideals? We shall need and sometime demand a substantial scientific foundation for a basis of dental training, and much of the material will be built on the basis of the so-called medical sciences, but it does not appear that as yet we are nearer to a medical degree than we were forty years ago. There is no doubt that medical practitioners have the wit and wisdom to determine with considerable accuracy the province of their part of the healing art, and they have already done this to a large degree. Medicine and surgery are not as closely related as surgery and dentistry though somewhat closely allied. We are intimately related in education, but shall we educate dental with medical students or as dental practitioners?

Dentistry has done much to make the people of the world comfortable and happy, and it has never failed to serve to the limits of its ability and with the best light at its disposal. We shall need more and more all the wisdom the world has to give us. We must have great teachers, scientists, and all the resources we can command to make our members resourceful as well as active in our related spheres of usefulness. We shall need for our work more and more the well trained minds of our boys and girls, that they shall develop minds that can think clearly and have bodies that are physically and mentally capable. Dentistry needs badly many young men and women who will train specially for dentistry. Dentistry actually needs in active practice today twice as many well trained dentists as we hope to have in many years. We very much doubt if we shall ever catch up with the world's needs. The expense of training dentists for the world is costly, and we fear the increased cost of preparatory training is destined to check the current of desirably trained dental students. We must properly prepare our students.

The demand is for a better educated class of dental students, and all dental teachers know that we can use better educated students as well as teachers. We realize that conditions are not ideal, but we are grateful that we have had hard working and self-sacrificing dental teachers who have made teaching a labor of love and not of aggrandizement. And we know, too, that until recently there has been no great assistance to encourage the teacher to do better things. We hope the state and philanthropy may work together to give us an educated faculty of medicine and dentistry. This will insure what the world needs most in the way of dental health. We really believe we are making more progress in scientific medical and dental science than we realize, or more at least than our critics think. Our college curriculum is being strengthened and our students do come to us better prepared than ever before. Give us stronger faculties, proper equipment, and better prepared students, and we shall meet the requirements amply.

---

**Index of Dental Literature** Under the auspices of the American Institnte of Dental Teachers there is being printed a complete index of the literature of dentistry. This will be the most useful contribution ever made to dentistry, and every effort is being made to have it accurate and satisfactory. It will be published in several volumes and as rapidly as the profession makes it practicable. Willing and adequate financial support is imperative. The first volume is now being printed. If you have any love for your profession's literature make your contribution at once, to Dr. A. Hoffman, 381 Linwood Ave., who is treasurer of the Institute. The price of the first volume will be six dollars.

